# A Minimal Theory of Creative Ability

**DOI:** 10.3390/jintelligence9010009

**Published:** 2021-02-16

**Authors:** Claire Stevenson, Matthijs Baas, Han van der Maas

**Affiliations:** Department of Psychology, University of Amsterdam, 1018 WS Amsterdam, The Netherlands; m.baas@uva.nl (M.B.); h.l.j.vanderMaas@uva.nl (H.v.d.M.)

**Keywords:** creativity measurement, creative potential, divergent thinking, intelligence, expertise

## Abstract

Despite decades of extensive research on creativity, the field still combats psychometric problems when measuring individual differences in creative ability and people’s potential to achieve real-world outcomes that are both original and useful. We think these seemingly technical issues have a conceptual origin. We therefore propose a minimal theory of creative ability (MTCA) to create a consistent conceptual theory to guide investigations of individual differences in creative ability. Building on robust theories and findings in creativity and individual differences research, our theory argues that creative ability, at a minimum, must include two facets: intelligence and expertise. So, the MTCA simply claims that whenever we do something creative, we use most of our cognitive abilities combined with relevant expertise to be creative. MTCA has important implications for creativity theory, measurement, and practice. However, the MTCA isn’t necessarily true; it is a minimal theory. We discuss and reject several objections to the MTCA.

## 1. Introduction

The creative act—from a toddler’s first building-block tower to Picasso’s Guernica—is a fascinating achievement of the human cognitive system. Creative ability is generally summarized into a short standard definition: the ability to produce original and useful products (Runco and Jaeger 2012), a definition that applies to all domains of creativity, from humor to the culinary arts and science to inventions. Creativity is an imperative skill the human race needs to solve global challenges such as climate change or privacy in a digital world. Organizations—from cancer research institutes to the fashion industry—are all looking for people with strong creative abilities (Ananiadou and Claro 2009; Casner-Lotto and Barrington 2006; IBM 2010). Creativity is therefore one of the most important 21st century skills educators want to instill upon their students (Henriksen et al. 2016). In order to select creative people or track creativity development good psychometric tests that reliably distinguish varying levels of creative ability (between people or over time) are a necessity. Unfortunately, existing instruments purporting to measure creativity often suffer from conceptual and psychometric shortcomings and do not meet the high requirements needed for personnel selection or measuring change (e.g., Baer 1994a; Barbot 2019; Montag et al. 2012; Said-Metwaly et al. 2017).

In our view, these seemingly technical issues have a conceptual origin. Creativity research lacks a clear formal model to understand and measure creative ability (Glăveanu 2012). In this paper, we propose a minimal theory of creative ability (MTCA), with creative ability being defined as people’s potential to achieve real-world outcomes that are both original and useful. MTCA describes which facets a theory of creative ability, at a minimum, must include. MTCA builds on earlier proposals for a modest conceptualization of creative ability (e.g., Baer 2012; Ericsson 1999; Runco 2009; Silvia 2015; Simonton 2003b). We reformulate these prior ideas into a more precise and concise form. We propose that, while many different variables are associated with creativity, only intelligence and expertise are essential for explaining and predicting individual differences in real-world creativity. The MTCA has important implications. It offers a parsimonious account for classic phenomena and findings in creativity research. It also provides clear guidelines for measuring creative ability and predicting creative achievements. The MTCA is in essence a minimal theory, and we discuss and dismiss possible variables and phenomena that could falsify it.

## 2. Positioning the MTCA

The MTCA is a theory based in differential psychology, the field Cronbach (1957) describes as the psychology of individual differences. The aim of differential psychology is to determine the nature, magnitude, causes, and consequences of psychological differences between individuals in the general population. So, with the MTCA we address how and why people differ in real-world creativity, where the major challenge is measuring creative ability. Please note that we focus on creative ability, not the creative process (for different accounts of the creative process see e.g., Gabora 2017; Hélie and Sun 2010; Kozbelt et al. 2010; Nijstad and Stroebe 2006; Schmidhuber 2010; for a thorough analysis of the study of individual differences versus processes see Cronbach 1957). So, the MTCA does not answer questions, such as, “How does the creative act take place?” and “What happens in the brain during creative problem solving?”. Instead, we focus on questions such as “What are the components of creative ability?”; “How can creative ability be measured?”; “Are individual differences in creative ability stable over time?”; and “Can creative ability be trained?”. We show that the MTCA provides clear answers to each of these questions about creative ability.

Because the MTCA is concerned with individual differences in creative ability, it is positioned in the Person perspective of creativity. The Person perspective represents one of the four general approaches to creativity research and focuses on which characteristics make a person creative (Rhodes 1961). With MTCA, we argue that intelligence and expertise are the essential person characteristics that distinguish between people scoring high or low on creative ability. The MTCA is also concerned with the Product perspective of creativity, focusing on the extent to which ideas, acts, and output are judged, often by relevant experts and stakeholders, to be creative (Amabile 1982; Montag et al. 2012; Simonton 2003c). Real-world creative products or outcomes are the most important criteria that the MTCA strives to predict. These creative outcomes may range from personal and everyday creative outcomes to eminent creative contributions (Boden 2004). For instance, they may refer to a sudden insight in how to solve a Sudoku (mini-c creativity); a new vegetable dish that your toddler enjoys eating (little-c creativity); a newly developed creativity test (Pro-c creativity); or a renowned paradigm-changing scientific theory (Big-C creativity). As such the MTCA addresses creative ability and potential at all levels, from mini-c to Big-C (Kaufman and Beghetto 2009).

The Process perspective, concerned with the cognitive processes that take place when someone is being creative, is not central to the MTCA. The MTCA only addresses research positioned in the Process perspective inasmuch as it says something about creative abilities. For example, we argue that many cognitive processes are involved in being creative and therefore cognitive ability is related to creative ability (e.g., Kozbelt et al. 2010; Simonton 2003b; Mumford and McIntosh 2017). Similarly, we only address the Press perspective, which investigates the circumstances or states that influence the expression of creative ability, to the extent that it informs us about creative abilities. For instance, environmental stressors, such as noise and cognitive load, make it harder for people to be creative because they hinder the execution of cognitive abilities (Byron et al. 2010; De Dreu et al. 2012). So, the MTCA does not deny that creative processes exist (it actually assumes their existence), nor that there are important situational constraints and triggers that influence how creativity emerges. However, the MTCA simply is not a theory about the creative process or environment, but about individual differences in creative ability (cf. Cronbach 1957; see e.g., Eysenck 1995, for a model in which both individuals and environmental factors interact to express creative achievements).

The MTCA is based on two key assumptions. The first assumption is that in order to be creative we use a wide variety of cognitive functions: functions that are generally assessed in intelligence tests. The second assumption is that creativity relies on the novel combination of existing knowledge and skills, i.e., expertise. Combined, these assumptions lay the groundwork for the MTCA, where intelligence and expertise are essential to understanding why people differ in creative ability. Below, we explore these assumptions further and conclude with a quasi formula for a minimal theory of creative ability.

**Assumption** **1.** *Creative ability requires many different cognitive abilities.*

The MTCA is based on the common assumption that we use a wide variety of our cognitive abilities to be creative (and probably tap all our cognitive functions across different tasks and settings). Whatever the challenge is, we use many cognitive abilities, such as those assessed in intelligence tests. For instance, we may use our perceptual abilities, conduct convergent and divergent thinking, search our memory for analogies, make use of our knowledge base, focus our attention, acquire new knowledge, etc. (e.g., Amabile and Pratt 2016; Beaty et al. 2014; Benedek et al. 2014; Kuncel et al. 2004; Newell and Simon 1972; Nijstad and Stroebe 2006; Sternberg et al. 2019).

We consider this assumption rather uncontroversial because most theories of creativity are consistent—or at least not inconsistent—with the idea that many cognitive functions are needed to be creative. For example, process theories of creativity are consistent when they describe the creative process in terms of consecutive stages (e.g., problem identification, idea generation, idea evaluation; see e.g., Basadur et al. 1982; Mumford and McIntosh 2017; Perry-Smith and Mannucci 2017; Wallas 1926) or some sort of cycle (e.g., including repetition and recursion) where different executive functions play a role (for overview see Kaufman and Glaveanu 2019; Kozbelt et al. 2010). This also applies to Amabile’s (1982) componential model and other componential theories that incorporate several creativity-relevant cognitive processes (e.g., breaking cognitive and perceptual sets, remembering accurately). We think typical cognitive process theories such as the Geneplore model (Finke et al. 1992) or Hélie and Sun’s (2010) Explicit–Implicit Interaction theory are also consistent with this assumption as they involve many cognitive functions. For instance, according to the Geneplore (generate–explore) model, people first retrieve existing elements from memory, form simple associations among these elements, and integrate, and transform them. The new ideas that result from these generative processes are then explored for their implications, checked against criteria and constraints and, if needed, refined (Finke et al. 1992).

Furthermore, theories on creativity that stress the importance of one cognitive function, such as associative thinking (Mednick 1962) or incubation (Sio and Ormerod 2009; Wallas 1926), are not inconsistent with our assumption as long as they acknowledge that other cognitive processes play a role in creativity as well. Inconsistency arises when a theory assumes that only one specific function is essential. This occurs when creative ability is reduced to divergent thinking ability only, for instance, as some psychometric theories of creativity propose (see Kozbelt et al. 2010).

**Assumption** **2.** *Creative ability requires expertise.*

We further assume that people apply their cognitive functions to analyze, combine, and integrate existing knowledge and skills to be creative (Simonton 2003b). This is why being creative in any domain also requires expertise in the domain at hand. Thus, the MTCA is based on the assumption that creativity always requires expertise (Baer 2011a, 2011b; Kim 2011a, 2011b; Plucker and Beghetto 2004). As with the “many cognitive abilities” assumption, we are certainly not the first, nor the only researchers to make this claim. A number of creativity theories recognize that creativity manifests in specific domains. For example, Baer and Kaufman (2005) use an amusement park metaphor to describe how domain specific creative abilities (e.g., writing sonnets) are related to broader and broader abilities (e.g., poetry writing, creative writing), where each level has its own requirements in terms of domain specific knowledge (e.g., word meanings) and skills (e.g., spelling, grammar, rhyming). Domain specificity also follows from Amabile’s (1982) componential model, where people use domain relevant skills to be creative and their motivation to be creative may be very specific to tasks within particular domains. Another example stems from Darwinian creativity models that argue that creativity involves a process of random generation and selective retention and elaboration of ideas (e.g., Campbell 1960; Simonton 1999b; but see Gabora 2017 for an alternative evolutionary model). According to these models, people’s brains produce (quasi)random variations of existing ideas that are part of a creator’s knowledge base, and the greater the knowledge base, the more potential new combinations that are truly creative are possible.

Other theories that discuss the domain specificity of creative output are, for example, Boden’s (2004) H-creativity or Big-C creativity (Kaufman and Beghetto 2009), which typically emerges by applying domain specific knowledge acquired over at least a decade. This is supported by archival evidence (e.g., Simonton 1991). For instance, Hayes (1989) discovered that for eminent composers at least 8 years of musical study were required before they wrote a masterwork, and the vast majority required at least 10 years. From the perspective of expertise development, practicing skills, engaging in activities, and enriching the knowledge base in a particular domain provide the necessary building blocks for creative work in that domain (Beghetto and Kaufman 2007; Ericsson et al. 1993; Simonton 2008; Weisberg 1999, 2018).

Although Big-C creative achievements in a particular field often require 10 years of expertise development in that field, there are notable exceptions. For instance, break-through inventions may open up new territory for accelerated discovery, sometimes resulting in entirely new domains. For instance, celestial objects and phenomena were suddenly observable with Galileo’s refinement of the telescope thereby explosively advancing the field of astronomy (Simonton 2012); and because microscopic organisms and cells could suddenly be explored with Van Leeuwenhoek’s single-lensed microscope, an entirely new domain, microbiology, came into being (Simonton 2012). However, even then, people build on their expertise (e.g., knowledge, scientific observation and reporting skills) to make discoveries and build and advance a domain.

## 3. A Quasi Formula for the MTCA

In sum, the MTCA assumes that being creative requires a variety of cognitive functions similar to those generally assessed in intelligence tests and expertise relevant to the domain at hand. Although there may be many factors that predict real-world creativity, we argue that only intelligence and expertise are essential for explaining individual differences in real-world creativity. The minimal theory of creative ability (MTCA) can be expressed by the following quasi formula:

C (Creativity) = I (Intelligence) × E (Expertise).

Thus, for a highly intelligent person without any expertise (in, say, culinary arts), C would be zero (in the domain of culinary arts). Furthermore, a resourceful and inventive craftsman without any formal education, perhaps even unable to read, may come up with creative solutions for problems in his or her area of expertise. Yet, creativity always requires some level of intelligent processing. The MTCA simply claims that whenever we are confronted with a creative task, we use a wide variety of cognitive abilities (e.g., our memory, our ability to reason by analogy, our visual–spatial skills, our metacognitive capacities, etc.) in combination with relevant expertise (e.g., experience with carpentry techniques, paint textures, conducting scientific experiments, etc.) to be creative. That is still a lot, but that is all there is. We argue that there is no special creative talent or faculty, nor is there a specialized (brain) area for being creative (e.g., Dietrich and Kanso 2010).

Just as with other theories, we suggest that creative ability constitutes a multiplicative relation of multiple factors. For instance, Eysenck (1995) defined creativity as a product of personality, environmental variables and cognitive ability, including intelligence and knowledge, whereas Simonton (1999a) and Jauk et al. (2013) used the multiplicative relation between multiple factors to explain skewed distributions in creative achievement. In the case of MTCA, the multiplicative relationship is limited to intelligence and expertise. The multiplication sign indicates that I and E both are necessary for creativity, but can also compensate for each other.

## 4. Phenomena Consistent with the MTCA

As a parsimonious framework of creative ability, the MTCA is consistent with a number of recurring phenomena in creativity research.

**Phenomenon** **1.** *Creativity is related to intelligence.*

Creativity research is fraught with evidence that intelligence is related to performance on commonly used tests of creative ability, such as divergent thinking, as well as to creative achievements (De Dreu et al. 2012; Jauk et al. 2013; Silvia 2015; Sternberg et al. 2019; see Sternberg and O’Hara 2000 for an in depth discussion of the relationship between intelligence and creativity.). For instance, Silvia (2015) estimates the correlation between IQ and creative ability as measured with divergent thinking tests to be *r* ≈ 0.50. In addition, tests of intelligence and executive functioning are robust predictors of creative eminence and actual creative achievements (Jauk et al. 2013; Karwowski et al. 2016; Silvia 2015; Simonton 2003a). The idea that cognitive abilities are strongly related is not new. Different subtests of IQ tests show a positive manifold of intercorrelations and different composite scores (factor analytic or sum scores) correlate very strongly (van der Maas et al. 2006). According to this perspective, cognitive abilities that in some creativity theories are considered special for creativity, such as divergent thinking, are simply part of a bigger construct: intelligence^1^. Although divergent thinking tests are not often part of IQ tests, we see no reason why valid divergent thinking tests with good test–retest reliability (e.g., with more items and automated scoring) shouldn’t be included. In fact, there have been attempts to make this happen (Kaufman et al. 2011; Süb and Beauducel 2005). We note that specific creative achievements may require a different balance of cognitive functions (Murphy 2017). Writing a poem requires more language abilities than solving a chess puzzle. However, because cognitive abilities are strongly correlated (van der Maas et al. 2006), and we use a wide variety of cognitive abilities to analyze, combine, and integrate existing knowledge and skills during creative problem solving, it is hardly surprising that general intelligence tests robustly predict real-world creativity.

**Phenomenon** **2.** *Creative achievement is domain specific.*

Most creativity tests correlate weakly among each other, i.e., have low convergent validity. This could partly be due to the low test–retest reliability (i.e., stability) of standardized creativity tests and resulting error variance (Barbot 2019; Cropley 2000). However, an even more important reason follows from the MTCA: creative achievements build on expertise, and because expertise is domain specific, creative achievements are also domain specific. Whatever the challenge, people simply have to work with the knowledge and skills that they have. An experienced sketch artist with good drawing skills may score higher on the figural subset of the Torrance Test of Creative Thinking (TTCT Torrance 2008) than an experienced creative writer, who, in turn, is more likely to score higher on the verbal subset of the TTCT and the Remote Associates Test (Mednick 1963). The MTCA thus also explains why many experts excel in their profession, but fail to find creative solutions for simple tasks in other domains (Kaufman et al. 2010a). More generally, the expertise component of MTCA clarifies the substandard construct validity of existing creative ability tests (cf. Baer 2012; Han 2003). A prime example of poor construct validity is that the figural and verbal components of the popular TTCT correlate < 0.10 (Baer 2011a, 2011b). Another example is that creativity scores from different domains (e.g., poetry and mathematics) tend not to correlate, or correlate only weakly (Baer 1994a, 1994b; Runco and Albert 1985; Simonton 2003b).

Domain specificity may also explain the low predictive validity of domain general creativity tests, such as the Alternative Uses Task (AUT Guilford 1967). Performance on such creativity tests generally correlates weakly with objective indicators of overt creative behaviors (Kim 2008; Zeng et al. 2011). For example, in a meta-analysis, Kim (2008) found a correlation of 0.22 between divergent thinking test scores and creative achievement. Note that for IQ, correlations with external criteria such as job success are reported to vary between 0.27 and 0.61 (for reviews see Schmidt and Hunter 1998; Sternberg et al. 2001) and predictive values of IQ as high as 0.81 are reported for educational achievement (Deary et al. 2007).

**Phenomenon** **3.** *Many experts have a few creative achievements; few experts have many.*

The distribution of achievements is often highly skewed in the population. For example, Murray (2009) describes this for professional golfers. Numerous players have won one or two tournaments, only four have won >30 tournaments, and then only one player (Jack Nicklaus) has won 71 tournaments (Murray 2009). This distribution is referred to as the Lotka curve, and also applies to creative achievements. This curve was first described by Lotka (1926) as a power law function where many authors produce a few publications, but only a few authors produce many publications (e.g., Simonton 2003c). Lotka’s curve has been found to hold for achievements in numerous domains, including chess and music composition (Murray 2009; Simonton 1999c). Many models have been proposed to explain this phenomenon (for overviews see Den Hartigh et al. 2016; Simonton 1999a). Generally, these models invoke some combination of factors, such as latent ability and number of produced outputs. In the MTCA creative ability is a combination of intelligence, which is generally assumed to be normally distributed in the general population, and expertise, which is either normally (in very selective samples) or exponentially distributed. Combining the normal distribution of intelligence and the skewed distribution of expertise results in a skewed distribution, which may explain why the distribution of creative achievements is also skewed (Den Hartigh et al. 2016; Simonton 1999a).

**Phenomenon** **4.** *Creative achievements across a career follow an inverted U-curve.*

Archival studies show how creative achievements unfold over the career of a creator, be it a scientist, composer, or painter (e.g., Ericsson 1999; Kozbelt 2008; Simonton 1997). What these studies tend to show is that the relationship between the number of creative achievements and career age follows an inverted U-shape, where the number of creative achievements of a creator steeply rises in the early decades of a career, then plateaus and slowly declines. The increase in creativity early in a career can be perfectly explained by expertise development, where the age curve for productivity appears to be a function of career age rather than chronological age (e.g., Khan and Minbashian 2019). Immersion in a domain over time leads to an increase in knowledge, activities, constraints, skills and procedures of a particular domain (Kozbelt 2008). By enriching the knowledge base in a particular domain, the creator has the necessary building blocks for creative work in that domain (Ericsson et al. 1993; Simonton 2008; Weisberg 1999, 2018). However, assuming that creators continue to develop their expertise across their career, why do their creative achievements not show a similar linear increase? Apart from extraneous factors that are beyond the scope of MTCA (e.g., successful scientists get managerial positions that limit their time to express their creative ability), the plateauing of creativity can be explained by how the acquisition of expertise follows an S-shaped curve: after a period of steep increase the acquisition of expertise levels off (Krampe and Charness 2018). That creativity ultimately declines may be caused by various extraneous factors such as poor health or changes in priorities or interest (Kozbelt 2008), but can also be explained by the fact that cognitive abilities, in particular what is generally considered as fluid intelligence, tend to decline during adulthood (Salthouse 2009). Simonton (1997) presented an endogenous explanation of these growth patterns. In future work an elaboration of the MTCA formula in such a dynamic model might be of interest.

**Phenomenon** **5.** *Limited effects of creativity training.*

Many forms of creativity training exist, most of which focus on the enhancement of a specific ability, such as divergent thinking or convergent thinking (Scott et al. 2004). Many studies show that creativity training effects are generally limited to the abilities that are specifically targeted and hardly generalize to real-world creativity (Baer 2012; J. M. Baer 1988; Scott et al. 2004). This lack of transfer makes perfect sense according to the MTCA. People rely, not on single, but on many cognitive abilities to turn domain specific expertise into real-world creative output. The same studies also show that creativity training effects tend to increase when creativity training is applied to exercises and examples within a relevant domain (Scott et al. 2004). This small, but nevertheless robust strengthening effect, also makes perfect sense according to the MTCA. Real-world creative output builds on domain specific expertise and relevant examples and exercises during training may help people to successfully apply newly acquired skills in their field of expertise.

**Phenomenon** **6.** *Absence of a specific neural basis of creativity.*

Decades of research exploring the neural correlates of creativity fail to show a specific brain area involved in creativity (Dietrich and Kanso 2010). If anything, this works shows diffuse prefrontal activation during the performance on creativity tasks (Dietrich and Kanso 2010). In line with this finding, more recent neural models of creativity include a large prefrontal network that is implicated in controlled memory retrieval and central executive processes (Beaty et al. 2016, 2018). This is much in line with the MTCA, which predicts that performance on creative ability tests relies on many cognitive abilities that underlie intelligence in general.

## 5. Implications

The MTCA has four important implications for creativity research and practice. First, because it relies on intelligence and expertise, creative ability has both a domain general component (i.e., intelligence) and a domain specific component (i.e., expertise) (e.g., Barbot et al. 2016; Plucker and Beghetto 2004). Because creative abilities depend on domain specific expertise, creative achievements are by definition domain specific. Baer (2012) suggests that, for each domain, we need “mini theories” about creative ability that specify the specific skills, knowledge and cognitive abilities that are required. Indeed, researchers probably need to specify the domain specific knowledge and skills that are relevant for a particular domain. For example, visuospatial abilities are probably more important in visual arts and architecture, whereas verbal comprehension abilities may be more relevant for poetry. Potentially, these specific cognitive abilities may have greater predictive value for creative performance in those specific domains, rather than an entire intelligence test (cf. Benedek et al. 2014). However, given the positive manifold of cognitive abilities, it may not make much difference which domain general abilities exactly are assessed as they are all highly correlated.

Second, because expertise and intelligence are essential components of creative ability, assessing individual differences in real-world creative potential should at the very least be done with tests of domain expertise and an IQ test. Creativity is ranked by managers among the most important skills of the 21st century (Ananiadou and Claro 2009; Casner-Lotto and Barrington 2006; IBM 2010), so if one wants to predict if a job candidate will be creative one should at the least test for expertise and intelligence. The good news, as explained below, is that assessing intelligence and expertise is rather straightforward; both have good psychometric properties, and can easily be implemented by an HR-team.

Third, training in domain general activities, such as divergent thinking, will not likely transfer to real-world creative achievements. Instead, creative achievements can only be enhanced by improving domain specific expertise (for recent discussions, see Ericsson and Harwell 2019; Macnamara et al. 2014). Whether intelligence can be trained or modified is another debate (Jaeggi et al. 2008), but domain specific expertise can certainly be developed. It is one of the main tasks of our educational system.

Fourth, although the MTCA is primarily concerned with individual differences in creative ability, it has implications for the environmental factors that constrain or facilitate creative achievements. On the one hand, a conducive environment, for example at someone’s work, can facilitate creative achievements (Amabile et al. 1996). On the other hand, situational factors that tax people’s cognitive abilities are expected to diminish people’s capacity for creativity. This is indeed what work on the effects of environmental stressors, such as noise and cognitive load, shows (e.g., Byron et al. 2010; De Dreu et al. 2012).

## 6. Objections and Limitations

There are several possible objections to, and limitations of, the MTCA. First, we accept the possibility that creativity is associated with other person characteristics such as positive affectivity, intrinsic motivation, or vulnerability to psychopathology (e.g., Amabile et al. 1996; Baas 2019; Baas et al. 2016; Hennessey 2019; Sternberg 2018). We do, however, propose that these characteristics are not essential factors for real-world creativity to emerge. For instance, someone may also achieve a creative feat while being generally grumpy, driven by dreams of glory and fame, and without psychopathological symptoms (Baas 2019; Baas et al. 2016; Byron and Khazanchi 2012). In addition, the correlations between these person characteristics and creative tests and achievements tend to be quite low. For example, the correlations with creativity for depressive mood (r = −0.06; Baas et al. 2019) and positive affectivity (r = 0.08; Baas et al. 2008) are close to zero.

Of all personality characteristics openness to experience is perhaps the single best predictor of creativity. Decades of research show that the personality trait openness to experience is related to divergent thinking (Gocłowska et al. 2019), distinguishes between scientists and artists that are low or high on creativity (Feist 1999, 2019), and predicts creative achievements across the lifespan beyond intelligence (Feist and Barron 2003). Moreover, the reported correlation of openness to experience with creative achievements is sometimes higher than that of intelligence (e.g., Carson et al. 2005; Jauk et al. 2013). Indeed, openness to experience is included in some creativity models (e.g., Eysenck 1995; Lubart and Guignard 2004). So, shouldn’t openness to experience, then, be included in a minimal theory of creativity? Again, we believe that openness to experience may not be essential. First, people can achieve remarkable creative feats by rather closed-mindedly exploring a narrow domain (Nijstad et al. 2010; Zabelina and Beeman 2013). Second, in many studies, including the one by Carson et al. (2005), university student samples were used. This greatly constricts the possible variance in intelligence of the sample without constraining variance in openness to experience, which, in turn, limits the effect sizes that can be obtained (Hunter and Schmidt 2004). In fact, using a somewhat more representative sample, Jauk et al. (2013) obtained structural equation models in which openness to experience was indirectly related to creative achievements through engagement in creative activities. It was intelligence—crucial to turning creative activities into creative achievements—that was the main predictor of creative achievements (Jauk et al. 2013). Third, further complicating things is that openness to experience is moderately related to intelligence and expertise. Openness to experience correlates with IQ scores (Harris 2004; Chamorro-Premuzic and Furnham 2006) and in some models of personality structure, intelligence and openness to experience are facets of a common factor (DeYoung 2006). In addition, with a preference for engaging in novel and varied experiences, people high in openness to experience are more likely to develop broad and rich expertise (Silvia and Sanders 2010). Before we can accept openness to experience as a third, crucial factor of creative ability, high-powered studies with a representative sample of the normal population are needed that would show that openness to experience significantly adds to the prediction of creative performance above and beyond Intelligence (I) and Expertise (E) and their interaction (I x E, also see the Discussion below).

Second, one may argue that the MTCA relies too much on expertise and intelligence as “can” factors, but misses out on motivation as the “want” factor. According to this perspective people, with all their cognitive abilities and expertise, achieve nothing if they are unmotivated to put their abilities to practice (Amabile 1983). First, we would like to remind the reader that the MTCA, based in differential psychology, is a theory to better understand individual differences in creative ability; i.e. “can” factors are key. We do not deny that motivational factors, such as persistence, interest, and passion, are important for the expression of creative ability in real-world creative outcomes (e.g., Amabile et al. 1996; Grohman et al. 2017; de Jesus et al. 2013). Furthermore, motivation is required to achieve expertise (Ericsson et al. 1993). However, motivation is volatile and easily influenced by environmental factors (e.g., Amabile et al. 1996). As such, rather than being an essential component of creative ability, motivation may be better studied from the Press perspective, research that is critical for understanding how the expression of creative ability is facilitated or constrained by the situation.

Third, the MTCA heavily rests on two concepts, intelligence and expertise, that also are not without their problems. The concept of intelligence is surrounded by controversies. It is, for instance, unclear whether intelligence is a unitary construct (Jensen 1998), a set of modules (Gardner 2011), or a network of interacting cognitive functions (van der Maas et al. 2006, 2017). However, in all these theories intelligence is a broad construct that captures a wide variety of cognitive functions, and that is also how it is measured. Furthermore, expertise, which refers to the characteristics of highly skilled and knowledgeable people “who are consistently able to exhibit superior performance for representative tasks in a domain” (Ericsson 2018, p. 14), is a research area with its share of debates (for instance, on the role of talent, Colvin 2011). Both concepts certainly require further clarification. However, there is one essential difference between these concepts and creativity: measuring general intelligence or specific expertise is much less problematic than the measurement of creative abilities, such as divergent thinking. Despite many limitations, IQ tests belong to the best tests produced by the field of psychology (Gottfredson 1997). Subtests of IQ tests show a positive manifold of intercorrelations (e.g., high convergent validity). Different composite scores (factor analytic or sum scores) correlate very strongly. Retest reliabilities of IQ (sub-)test scores are high, and criterion validity (in terms of the prediction of scholastic success) is also high (e.g., Deary et al. 2007). As expertise is domain specific by definition, generally applicable expertise tests do not exist. Yet, domain specific expertise can often be tested with existing tests, such as college exams or exams used for certifications. In a famous area of expertise research, chess, van der Maas and Wagenmakers (2005) found that different measures of chess ability show the same positive manifold of correlations that intelligence subtests do. In addition, these measures were excellent predictors of chess performance (Elo ratings). Tests of domain expertise of good quality exist for numerous scholarly domains (e.g., GRE Subject tests; Powers 2004). While objective tests of creativity in artistic domains are problematic, constructing good tests of technical skills in these domains appears far less challenging (e.g., Chan and Zhao 2010; Law and Zentner 2012). Given the narrow set of skills to be assessed in a test of expertise, it is relatively easy to meet the high psychometric standards required for measuring individual differences.

Fourth, expertise and general intelligence are not entirely independent constructs. On the one hand, the definitions of crystallized intelligence and expertise overlap somewhat. On the other hand, we would expect an interaction between expertise and general intelligence because intelligence plays a role in developing expertise (Deary et al. 2007). For example, a minimum level of general intelligence is clearly required to reach high levels of expertise in, say, music composition. However, this developmental dependency is only strong in the early stages of skill acquisition; in later stages the relationship between intelligence and level of expertise breaks down (Krampe and Charness 2018). For example, chess performance (Elo ratings) can be predicted in part by a player’s intelligence, but chess experience remains the strongest predictor of attained skills (Grabner et al. 2007). We note that for many studies that fail to show a significant correlation between intelligence and creative performance or between intelligence and expertise, the sample is restricted to experts (Bayer and Folger 1966; Cole and Cole 1973). The restriction of range in a key variable is an important methodological limitation. This restriction of range can of course also occur in practice. Suppose one wants to hire a full professor. The variation in IQ and expertise of the sample of candidates might be very small, such that these measures are not informative. Luckily, in these very restricted cases, one can consider creative achievement directly. In less restricted samples, for college admission for instance, we expect added value for both IQ and expertise measures.

Fifth, the MTCA does not incorporate divergent thinking as a separate critical component of creative ability. We certainly consider divergent thinking as part of MTCA, but then as part of the many cognitive abilities that support creativity. Thus, as discussed under Phenomenon 1, it would fall under Intelligence. Divergent thinking tests are not often part of standard IQ tests (Kaufman 2015). However, we do not see why a valid and reliable test of divergent thinking could not be included in intelligence assessment because, in our view, it falls within the positive manifold of cognitive abilities that IQ tests purport to measure (cf. Silvia 2015). Some study results suggest that incorporating divergent thinking may increase the predictive validity of intelligence tests (Kim 2008; Plucker 1999).

Sixth, and finally, it could be argued that MTCA focuses too much on Pro-c and Big-C creativity (e.g., scientists developing a new creativity test, people that have received a Nobel prize) rather than one’s personal and everyday creative insights (mini-c and little-c creativity, e.g., a child who endearingly impersonates a firefighter with imaginary attributes; Boden 2004; Kaufman and Beghetto 2009). Indeed, the creative process hardly differs for different levels of creativity. However, rich and strong expertise within a particular domain may be more critical for Pro-c and Big-C creative achievements within that domain (Kaufman and Beghetto 2009). However, one also needs some expertise for mini-c and little-c. For example, when impersonating a firefighter, you need to know which attributes belong to a firefighter and use creative problem solving to find original alternatives within your reach. So, with mini-c and little-c creativity intelligence may weigh in more than expertise, but, still, both I and E are required.

A possible limitation is that the MTCA ignores that creative achievements often, and increasingly, emerge in social networks (Perry-Smith and Mannucci 2017). Creators may greatly benefit from the expertise of others that may provide the missing link of a puzzle that creators are working on (Hargadon and Bechky 2006; Johnson 2010). As a consequence, person characteristics that facilitate help seeking and giving may be important for creativity to emerge in social settings. Still, the importance of expertise and intelligence is upheld. Once shared, the expertise of others becomes part of one’s own expertise. Although people may certainly borrow the expertise of others, people still need the cognitive abilities to process, assimilate, develop, and integrate the knowledge and perspectives that others offer (Nijstad and Stroebe 2006).

Another limitation is that MTCA primarily focuses on the standard definition of creative achievement, which refers to recognized outcomes that are both novel and useful (e.g., Montag et al. 2012; Runco and Jaeger 2012). However, sometimes other creativity criteria are added, including surprise (Simonton 2012) and aesthetic value and authenticity (Kharkhurin 2014). How flexible is MTCA in dealing with such additions to the standard definition? Indeed, MTCA can also help to explain variance in creative outcomes when other dimensions or criteria are considered. For instance in Kharkhurin’s conception of creativity, aesthetic value is about the content and the techniques of an (artistic) work and an outcome is high on aesthetic value when it presents the fundamental truth of nature, strives to arrange expressive elements in a perfect order, expresses the essence of the phenomenal reality in an efficient manner, and is sufficiently complex. Using their expertise and cognitive abilities, people analyze and explore a particular phenomenon to better capture its essence and using their expertise people give content and context to an idea and apply skills and techniques. This is, for instance, what artists do when preparing a work of art and then using their skills (e.g., painting techniques) and knowledge (e.g., about material) to create an art work. Authenticity refers to creative work that expresses a creative person’s inner self (Kharkhurin 2014). Obviously, people need self-knowledge (expertise) to make an authentic work. However, this aspect of creativity may not be assessed by independent stakeholders/experts and, as a personal judgment, would fall in the mini-c level of creativity.

## 7. Discussion

With the Minimal Theory of Creative Ability (MTCA) we contend that intelligence and expertise are the only necessary components of the ability to be creative, the ability to produce novel and useful ideas or products (e.g., Montag et al. 2012; Runco and Jaeger 2012). The MTCA builds on previous theories, models, and findings in creativity research and offers five important contributions. First, MTCA is carefully cast in an individual differences (as opposed to a process) approach (cf. Cronbach 1957), thereby advancing conceptual clarity. Second, MTCA is a formal theory that is both simple and falsifiable, paving a path for further rigorous creativity research. Third, we compiled a comprehensive list of six robust phenomena in creativity research that any theory of creative ability should account for—and MTCA does. Fourth, we discuss six possible objections to MTCA and refute these. Fifth, MTCA has an important implication for measuring creative ability because MTCA simply reduces creative ability to just two other measurable concepts. We claim that creative ability can best be measured with a combination of an intelligence test and tests of expertise, potentially solving the problems with measuring individual differences in creative ability and predicting real-world creative achievements.

We call the MTCA a minimal theory on purpose. It is not that we believe the MTCA is necessarily true. With a minimal theory, we propose a Popperian challenge: Can we falsify the MTCA? There is a vast amount of creativity research, which may well provide strong evidence against the MTCA. Rejections of MTCA could involve new research, but also reviews of existing research lines. In all cases we think methodological rigor is required. Creativity research will benefit from strict methodological research practices. This includes using instruments with known psychometric properties, sufficient study power, a representative sample of the normal population, and preregistration of studies (de Groot 2014; Nosek et al. 2015), and using theory testing rather than discovery based research (Oberauer and Lewandowsky 2019).

We see at least four ways in which the MTCA could be rejected. First, there is possibly more to creativity than the combination of intelligence and expertise. This requires a differential psychological study showing that some stable individual differences factor (e.g., a personality trait, divergent thinking ability, etc.) significantly adds to the prediction of creative performance above and beyond Intelligence (I) and Expertise (E) and their interaction (I × E). However, existing research faces many methodological problems (e.g., the poor test–retest reliability of divergent thinking tests, the restriction of range in intelligence in WEIRD study samples). Thus, showing that individual differences in real-world creativity require more than the combination of intelligence and expertise probably needs new empirical research that adheres to the strict methodological research practices outlined above.

Second, the MTCA could be rejected if we can somehow demonstrate that creativity is domain general. One way is to argue that individuals commonly display creativity in many diverse domains. However, the polymath or Renaissance man is uncommon (Cassandro 1998; Kaufman et al. 2010b; Ma and Uzzi 2018). Indeed, versatility, achieved by developing multiple areas of expertise, rather than solely focusing on one domain, may aid creative achievement (Simonton 2000). Furthermore, creativity in very different domains is not ruled out by MTCA as long as one has expertise in each domain (see also Simonton 2003b). A better way to falsify the MTCA by demonstrating that creative ability is domain general would be to develop a reliable and valid test of domain general creativity with predictive power comparable to IQ tests (so high predictive validity) and also good convergent and discriminant validity. Perhaps two recently published creativity test batteries, the Evaluation of Potential Creativity (Lubart et al. 2011) and the Runco Creativity Assessment Battery (Creativity Testing Services 2011), will meet these high psychometric standards. Similarly, major improvements in creativity assessments that rest on divergent thinking could be used to falsify the MTCA, where creativity could then be considered equal to divergent thinking, C = DT, rather than C = I × E.—although, as mentioned above, it is very possible that divergent thinking is just missing from standard IQ tests.

Third, if valid measures of creative ability within domains are available, several tests of the MTCA’s domain specificity assumption are possible. Assessing actual creative performance using panels of expert judges with the Consensual Assessment Technique (Amabile 1982; Baer and McKool 2009; Myszkowski and Storme 2019) may be useful. By using expert ratings to assess creative achievements (C) we can test how much variance is explained by I, E and I × E and whether additional variables significantly help predict C. If we have expert ratings of C in different domains, we could also test whether the residuals of the regression of I, E and I × E on C correlate. This would suggest domain generality. Another research line might concern the threshold hypothesis, which says that the linear relationship between C and I disappears above some level of I (e.g., Barron 1963; Torrance 1962). The evidence on this effect, however, is mixed (Jauk et al. 2013; Karwowski et al. 2016; Preckel et al. 2006). Again, we stress the importance of using a ‘good’ test of C, along with sufficient variance in intelligence. A low correlation between an IQ measure and some measure of C, with a low test–retest reliability, is not a rejection of the MTCA (e.g., Wallach and Kogan 1965), nor is a low correlation between IQ and C when there is restriction of range in IQ scores.

Fourth, if creativity improves for some reason without increasing expertise or intelligence then we could reject the MTCA. For example, in the MTCA domain general training of creativity is impossible (see Baer 2012 for a similar point). According to MTCA it is only possible to improve creative ability by improving general cognitive functions (such as working memory) or improving domain specific expertise. Thus, the MTCA could be rejected by demonstrating that domain general creativity training improves creativity without improving an aspect of intelligence (Scott et al. 2004). Similarly, MTCA predicts that creativity improves with age due to increasing expertise. So, if we can prove that younger children are more creative than older children (German and Defeyter 2000), adolescents are more creative than adults (e.g., Stevenson et al. 2014), or that creativity does not improve with age (prior to cognitive decline) (e.g., Simonton 1977, 1997), then we could reject the MTCA. A related prediction is that creative ability improves, rather than declines, with increased expertise. So, if cultivating expertise consistently leads to less creative performance, such as “habitual” performance or entrenchment, then we would have arguments against the MTCA (Dane 2010; Ford 1996; Weisberg 2018).

## 8. Conclusions

The MTCA states that creative ability within domains is essentially due to the combined effects of intelligence and expertise. To assess creative ability, one simply needs to measure intelligence and domain specific expertise to determine a person’s ability to be creative within a given field. If MTCA holds, then the construction of valid domain general creativity tests is doomed to fail.

## Footnotes

1 Sometimes, divergent thinking performance is seen as an indicator of everyday creative achievement (little-c creativity; see e.g., Kaufman and Beghetto 2009). In our view, divergent thinking cannot be treated as the criterion variable. Instead, it should be seen as a cognitive ability that predicts real-world creative achievements (Karwowski et al. 2016; Runco and Acar 2012). Going one step further, we see divergent thinking as one of the many cognitive abilities, such as memory search and retrieval or analogical reasoning, involved in creativity that fall under the umbrella of intelligence.
